# Correlation in gene expression between the aggravation of chronic obstructive pulmonary disease and the occurrence of complications

**DOI:** 10.1080/21655979.2020.1839216

**Published:** 2020-10-27

**Authors:** Yuchen Cai, Runhan Liu, Xinhe Lu, Qiming Zhang, Xinwei Wang, Huijing Lian, Haohua Wang

**Affiliations:** a Department of Mathematics, School of Science, Hainan University, Haikou, Hainan Province, China; b School of Life and Pharmaceutical Science, Haikou, Hainan Province, China; c School of Electrical and Information Engineering, Anhui University of Science and Technology, Huainan, Anhui Province, China; d College of Information Science and Engineering, Ocean University of China, Qingdao, Shandong Province, China; e School of Economics, Hainan University, Haikou, Hainan Province, China

**Keywords:** Chronic obstructive pulmonary disease, bioinformatics, complication, shared gene

## Abstract

Aggravation of the chronic obstructive pulmonary disease (COPD) often leads to a slew of complications, but the correlation between COPD aggravation and the complications on the basis of molecular level remains unclear. In this study, gene expression profiles of COPD in patients at early and aggravation stages were collected and differentially-expressed genes were selected. Meanwhile, gene expression data implicated in COPD complications were analyzed to establish a regulatory network of COPD aggravation and COPD related complications. In addition, the gene enrichment function of DAVID6.7 was utilized to evaluate the similarities between COPD aggravation and COPD complications in term of biological process. By analyzing the genes of COPD aggravation and the COPD complications, we found 18 genes highly related to COPD aggravation, among which haptoglobin (HP) was correlated with 14 complications, followed by ADRB2, LCK and CA1, which were related to 13, 11 and 11 complications, respectively. As far as the complications concerned, obesity was regulated by 17 of the 18 genes, which indicated that there was a close correlation between COPD aggravation and obesity. Meanwhile, lung cancer, diabetes and heart failure were regulated by 15, 15 and 14 genes, respectively, among the 18 selected genes. This study suggested the driver genes of COPD aggravation were capable of extensively regulating COPD complications, which would provide a theoretical basis for development of cures for COPD and its complications.

## Graphical Abstract

**Figure:**
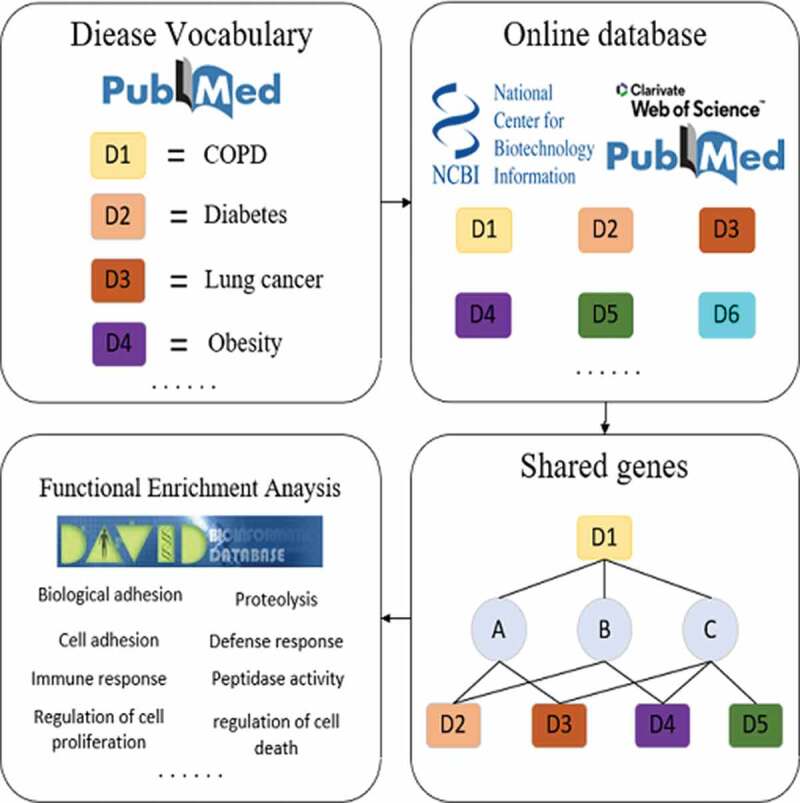

## Introduction

Chronic obstructive pulmonary disease (COPD) features chronic bronchitis. It is a disease caused by airflow obstruction and is attributable to cigarette smoking. Since 2010, it has been the third leading cause of deaths in the world [1], and its death toll has been rising year by year [2]. COPD is also one of the main burdens on the public health system in China [3], with an average COPD incidence higher than 8% among residents aged 40 and above. COPD has a long pathogenesis which usually features an ongoing and incompletely reversible development. It causes lung failure, pneumothorax and dyspnea and requires regular medication. If not treated in time, COPD will deteriorate into lung failure, spontaneous pneumothorax and heart failure. Worse still, critically-ill patients with COPD often suffer a high mortality rate because the disease usually turns irreversible in the late stage. Existing data show 50% of COPD aggravation cases are caused by respiratory infection, 10% of which are attributed to environmental pollution (dependent on the season and geographical location), while 30% to 50% result from unknown causes [4]. At present, the screening of COPD candidate genes is often through meta-analysis or genome-wide association studies, and these methods have identified some genomic associations between COPD and lung cancer.Hence, seeking significant clinical indicators from patients in the early stage of COPD aggravation will deepen our understanding of the causes and development of COPD and its aggravation.

COPD complications are defined as concurrent diseases, and their incidence is higher among the COPD/COPD aggravation patients than among healthy individuals. Besides, it has a marked impact on the treatment or prognosis of COPD patients. For instance, COPD patients are susceptible to diseases such as cardiovascular disease, myasthenia, lung cancer, osteoporosis and metabolic syndrome [5–7], which usually increase the incidence of acute COPD aggravation, affect the patients’ regular life and reduce their chance of survival. The pathogenesis of COPD complications remains unknown, but the chronic inflammation of COPD has been proved to be likely to cause some complications [8]. Currently, the screening of COPD candidate genes is often carried out through meta-analysis or genome-wide association studies, and these methods have identified some genomic associations between COPD and lung cancer, but there are still great challenges for the treatment of COPD and its complications, such as how to strictly control bronchitis reactions through medication. This can reduce the risk of COPD complications, but the inflammation alleviation rate of COPD remains low. Controlling inflammation alone cannot effectively reduce the danger of COPD complications.

Is there a causal or coincidental relationship between COPD complications and COPD development? What factors play a role in this relationship? These are the questions to be answered. COPD shares the same pathogenesis with many concurrent diseases. By analyzing the driver genes of COPD pathogenesis and those of COPD complications, this study aims to find out if the concurrent diseases are caused by the complications or by COPD, with the hope of contributing to the prospective multi-target and multi-channel intervention in the occurrence and development of diseases and the treatment of COPD and its complications. This study selected key driver genes of COPD complications with the gene regulation network and analyzed how the abnormal expression of COPD aggravation genes resulted in the complications, thus shedding a new light on the pathogenesis, genetic relationship and key targets of this disease to benefit the design of drugs and therapies. With information collected about 16 complications related to COPD from websites such as NCKI and PubMed, this paper delved into the molecular biological relationship between the complications and COPD aggravation. The pathogenesis of COPD and its complications is yet to be explored, but it is certain that the factors and mechanisms shared by the two may play a potential role in their correlation. A new relationship between COPD and diseases like obesity and diabetes was established in the analysis of these genes. Ultimately, this study adopted DAVID6.7, an online analysis tool for gene enrichment, and utilized bioinformatics to further evaluate the connection in biological processes between COPD and its complications. This will facilitate further research on the role that COPD complications play in COPD aggravation. In summary, this article uses online databases and online analysis tools to pinpoint the key genes of COPD aggravation and its complications, thereby providing a theoretical basis for development of cures for COPD and its complications.

## Materials and methods

### Collection of gene expression profiles of COPD

The differentially-expressed genes were obtained from NCBI. The first step was to input ‘COPD’ and ‘COPD aggravation’ as keywords onto the search engine of the GEO database. After the data sets that were inconsistent with the research topic were removed, three data sets of COPD patients in the early stage and one data set of COPD in the aggravation stage were kept. The following keyword combinations were searched for on PubMed: COPD/chronic obstructive pulmonary disease aggravation and gene; chronic obstructive pulmonary disease/chronic obstructive pulmonary disease aggravation and differentially-expressed gene; chronic obstructive pulmonary disease/chronic obstructive pulmonary disease and gene expression profile. This method was also adopted to further select differential genes from Web of Science and China National Knowledge Infrastructure (CNKI). From the collected COPD data set, 2000 genes with a fold change difference greater than 5 were randomly selected as the sample data set for gene comparison and enrichment analysis.

### Collection of gene expression profiles of COPD complications

To identify some common COPD complications, this study searched the following keyword combinations on PubMed: chronic obstructive pulmonary disease/chronic obstructive pulmonary disease aggravation and complication; COPD/COPD aggravation and complication. Then, the correlation between the abstracts and the objectives was evaluated, and relevant articles were selected.

To obtain more information about the types of COPD complications, this study conducted a search in the following categories: metabolic diseases, respiratory system diseases, cardiovascular diseases, and musculoskeletal diseases. The keyword combinations for the search were as follows: chronic obstructive disease/chronic obstructive pulmonary disease aggravation and metabolic disease; chronic obstructive disease/chronic obstructive pulmonary disease and respiratory system disease; chronic obstructive disease/chronic obstructive pulmonary disease aggravation and cardiovascular disease; chronic obstructive disease/critical chronic obstructive pulmonary disease aggravation and musculoskeletal disease. Relevant articles were chosen according to the assessment of the retrieved article abstracts. For the retrieved articles with a strong correlation, ‘Related citation’ on PubMed was utilized to acquire more related literature. The same method was applied on Web of Science and CNKI to retrieve relevant literature. The correlation of all the retrieved abstracts was evaluated to select the articles connected with this study.

To gather as many gene expression profiles of COPD complications as possible, this study undertook a well-rounded assessment of the literature about COPD/COPD aggravation and COPD complications, and then chose 16 most common COPD complications for the follow-up research. Later, an attempt was made to obtain the in-situ tissue or peripheral blood mononuclear cell (PBMC) gene expression profiles of these 16 complications from NCBI’s GEO database and compare them with normal samples to analyze the differential genes. After that, the standards of selecting differentially-expressed genes were made as follows: precedence was given to the differentially-expressed genes from PBMCs; for the complications with a stronger correlation with COPD/COPD aggravation and a larger number of differential genes, the P value was set as lower than 0.005; for the ones with a smaller quantity of data in the GEO database, the P value was set as lower than 0.05.

### Statistical analysis

DAVID6.7 and DEG were employed for the annotation and analysis of the biological genes. Data of COPD genes were randomly selected three times for analysis, and the average value of three parallel tests was taken. When comparing the number of biological processes, biological processes involving less than three genes should be eliminated.

The following method was used to select highly expressed genes related to COPD comorbidities: search for the gene name in PubMed, and if there are literatures that the expression of a gene is directly involved in the regulation of COPD comorbidities, this gene is considered to be strongly related to COPD comorbidities. If there are literatures that do not clearly indicate that the expression of this gene is directly involved in the regulation of COPD comorbidities, but there are many similarities between the biological processes involved by genes and this disease, such as involvement in cell apoptosis, inflammation, etc., then the gene is considered to be slightly related to COPD comorbidities. Otherwise, the gene is considered unrelated to the disease.

## Results

### Differentially-expressed genes in the early and aggravation stages of COPD

40,314 healthy differential genes in the early and aggravation stages were obtained from online databases including GEO, PubMed, Web of Science and CNKI. Then, 600 differential genes with the greatest change were selected, including 200 obtained from the comparison between the early stage of COPD and the healthy state, 200 from the comparison between the aggravation stage of COPD and the early stage, and 200 from the comparison between the aggravation stage of COPD and the healthy state. Out of 600 genes, 18 potential genes highly related to COPD aggravation were selected.

### Genes implicated in COPD complication

By reviewing the literature, the study selected 16 COPD complications with the highest incidence (Table 1), including diabetes, metabolic syndrome, obesity, myasthenia, osteoporosis, pulmonary hypertension, sleep apnea, lung cancer, heart failure, atrial fibrillation, myocardial infarction, ischemic heart disease, stroke, depression, cachexia and anemia.The reasons for choosing these 16 diseases are as follows: on the one hand, there are many literatures showing that these diseases are the most common comorbidities in COPD patients,and on the other hand, it is difficult to collect gene expression profiles on other complications of COPD. Metabolic syndrome and osteoporosis ranked top among these complications. 109 groups of genes were obtained through stratified sampling from the GEO database. The differentially-expressed genes related to COPD complications were chosen, and their quantity was shown in Table 1. The number of differential genes related to heart failure was the largest (1968), while that of differential genes related to sleep apnea was the smallest (882). Differential genes from each complication were compared to the differential genes of COPD aggravation which were twice more in quantity (the COPD aggravation genes were randomly obtained from the above databases and the quantity of the shared differential genes was shown in Table 1). To some extent, the quantity of shared genes reflects the degree of genetic correlation between the complications and COPD aggravation. The results revealed that lung cancer, diabetes, pulmonary hypertension and obesity were the four complications with the strongest correlation with COPD aggravation, and the number of shared genes was 217, 217, 212 and 195, respectively. Correspondingly, the genes shared by these four complications and COPD aggravation accounted for the highest percentage in the complication genes respectively. This indicates that there was a strong correlation in the pathogenesis of gene regulation between these four complications and COPD aggravation. Metabolic syndrome, depression and sleep apnea were the three complications which shared the least genes with COPD aggravation. These shared genes might cause complications by regulating COPD aggravation or lead to COPD aggravation through complications.

**Table 1.** The number of differentially-expressed genes of COPD complications and the number of genes shared by COPD complications and COPD aggravation

Category	Disease	Incidence (%)	Number of Original Genes of Complications	Number of Genes Shared with COPD Aggravation	Percentage of Shared Genes in the Original Genes of Complication
Nutrition and metabolic diseases	Diabetes	23–27	1902	217	11.4%
	Metabolic syndrome	45–55	1094	58	5.3%
	Obesity	25–33	1771	195	11.0%
Musculoskeletal diseases	Myasthenia	25–30	-	-	-
	Osteoporosis	30–35	1593	70	4.4%
Respiratory diseases	Pulmonary hypertension	20–25	1675	212	12.7%
	Sleep apnea	4–7	882	34	3.9%
	Lung cancer	15–20	1873	217	11.0%
Cardiovascular diseases	Heart failure	12–15	1968	154	7.8%
	Atrial fibrillation	20–23	-	-	-
	Myocardial infarction	7–11	1618	106	6.6%
	Ischemic heart disease	16–20	-	-	-
	Stroke	15–18	1776	148	8.3%
Others	Depression	15–20	1973	81	4.1%
	Cachexia	8–10	1307	93	7.1%
	Anemia	10–15	1580	138	8.7%

Table 1 shows some information about COPD complications, including the incidence of the diseases, the number of genes related to the diseases, the number of genes shared by the diseases and COPD aggravation, and the percentage of shared genes in the original genes. The incidence of the diseases was confined to certain intervals according to databases like PubMed and Web of Science, and the gap between the upper and lower limits was no higher than 5%.

### Genes mediated in crosstalk of COPD aggravation and complications

**Figure 1.**
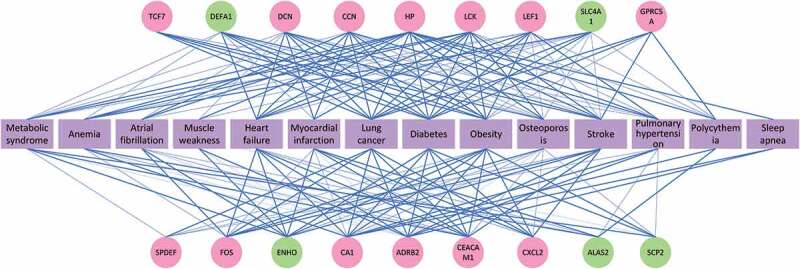
Correlations between the 18 selected genes and COPD complications

Meanwhile, it was found that the 18 genes highly correlated with COPD aggravation were related to at least three COPD complications. The degree of the correlations between the selected 18 genes and the complications is shown in Figure 1. The network shows the relevance in gene between the complications and COPD aggravation, and a thicker blue line implies stronger relevance. Of these 18 genes, 13 (in the red circles) are noticeably relevant to the COPD pathogenesis. The remaining five (in the green circles) showed abnormal expression in COPD/COPD aggravation, although their connection to the process remains to be confirmed. In particular, heptoglobin (HP) was correlated with 14 complications and was the gene connected to the largest number of complications. HP was followed by ADRB2, LCK and CA1, which were related to 13, 11 and 11 complications, respectively. As far as the complications are concerned, obesity was regulated by 17 of the 18 genes, which indicates that there was a close tie between COPD aggravation and obesity. It was followed by lung cancer, diabetes and heart failure, which were regulated by 15, 15 and 14 genes, respectively. The results were also related to the number of genes COPD aggravation shared with obesity, lung cancer and diabetes respectively. This further revealed that these complications were strongly related to COPD aggravation molecularly.

### The biological path linking COPD aggravation and complications

Figure 1 shows the degree of relevance between the selected 18 genes and the complications: This network reveals the correlation between the diseases and the genes, and a thicker blue line indicates a higher degree of relevance. Two colors, pink and green, were utilized to represent these 18 genes: a pink gene means that there is a proven significant correlation between it and the COPD pathogenesis; a green gene implies that it has not been proved to be the pathological gene of COPD/COPD aggravation but it showed an abnormal expression in COPD/COPD aggravation. A purple complication is a COPD-related one selected by clinical experts. There were 14 complications in total.

**Figure 2.**
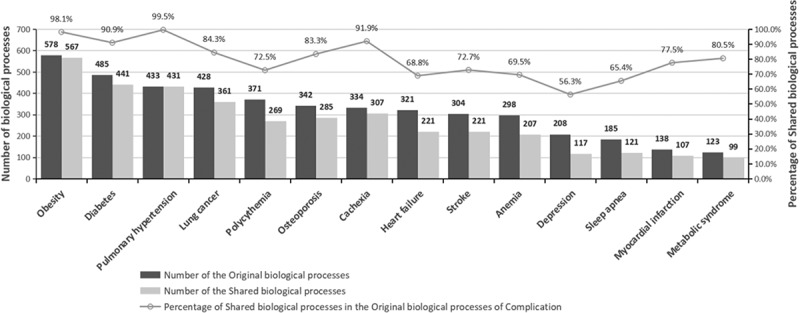
Similarities between COPD aggravation and COPD complications in biological processes

Figure 2 shows the degree of relevance in biological processes between COPD aggravation and complications, indicating information on the number of the original biological processes, the number of the shared biological processes, and the percentage of shared biological processes in the original biological processes of complication. Of these complications, obesity had the largest number of shared biological processes, followed by diabetes, pulmonary hypertension and lung cancer. In addition, the top four complications in terms of the percentage of shared biological processes in the original ones were pulmonary hypertension (99.5%), obesity (98.1%), cachexia (91.9%) and diabetes (90.9%).

Based on gene enrichment analysis, Figure 2 shows the biological correlation between COPD aggravation and complications. In this study, the biological paths involving at least three genes were selected, and the number of the biological processes related to each COPD complication is shown in Figure 2. Specifically, obesity shared 567 biological processes with COPD aggravation, being the complication sharing the largest number of biological processes with COPD. It was followed by diabetes, pulmonary hypertension and lung cancer, which shared 441, 431 and 361 biological processes with COPD respectively. Interestingly, the four complications sharing the largest number of biological processes with COPD aggravation were the ones sharing the largest number of genes with COPD aggravation. This further indicates the correlation in gene expression that COPD shared with obesity, lung cancer, diabetes and pulmonary hypertension. Moreover, the percentages of the biological processes that the complications shared with COPD aggravation in all of the complication’s original biological processes were calculated. The results show that the complications with the highest percentage were pulmonary hypertension, obesity, cachexia and diabetes. This coincides with the percentage of shared genes in the original genes of the complications, as shown in Table 1. In addition, metabolic syndrome shared fewer biological processes with COPD than other complications, but the shared biological processes occupied a high percentage of the original biological processes of metabolic syndrome. This demonstrates that there may be a stronger correlation between metabolic syndrome and COPD aggravation.

## Discussion

As a severe incident, COPD aggravation has a high fatality rate and would prompt a series of complications. By analyzing the genes involved in COPD aggravation and complications, this study found a genetic correlation between the two. The findings show that HP, ADRB2, LCK and CA1 could extensively regulate COPD complications. Obesity, lung cancer, diabetes and pulmonary hypertension were the four complications that correlated the strongest with COPD aggravation. Emphasis will be placed on how the major genes of COPD aggravation regulate the occurrence of the complications. In this way, it is hoped that the findings of this study will provide a theoretical basis for the prospective multi-target and multi-channel intervention in the occurrence and development of diseases and the future treatment of COPD and its complications.

In the study, HP was found to be involved in the occurrence of 14 COPD complications. The HP in blood plasma originates from the lungs, the second main organ where HP forms [9]. Lung HP works in immune adjustment in the inflammatory process of the respiratory system, protecting the lungs from inflammatory injury caused by the inflammation mediator [10]. COPD is a disease rising from inflammation and involving many cytokines and mediums. It can cause systematic inflammatory reactions, including the activation of C-reactive protein (CRP), IL-6, fibrinogen, leukocyte, and tumor necrosis factor (TNF)-α3, thereby markedly increasing the HP of the lung tissues and peripheral blood of COPD patients [11–13]. HP is an acute phase protein, and consequently, it would rise more significantly in COPD aggravation. Meanwhile, some studies have shown that the HP level correlates positively with systemic adverse reactions and oxidative stress of COPD patients [14]. These are also consistent with the results of this study. Besides, HP deficiency would injure lung tissues, thus triggering emphysema and COPD. To a large extent, HP can reflect the inflammatory reaction and severity of COPD [10] and is thus strongly related to COPD complications.

In this study, ADRB2 was correlated with 13 COPD complications. Responsible for coding the β-2-adrenergic receptor on the surface of cells, it plays a role in the relaxation of smooth muscles and in the dilation of airway smooth muscles, protecting the lungs from long-term bronchospasm [15]. A damaged ADRB2 may result in a narrower airway and increase vulnerability to COPD [16,17]. Besides, it can reduce mucosal lesions by promoting the conveyance of microvilli of bronchial epithelial cells [18,19]. Meanwhile, ADRB2 has been reported to be involved in cardiovascular diseases (heart failure and myocardial infarction), diabetes, lung cancer and obesity [20–23]. A similar relationship between receptor expression and function is also found in vascular myocardium [24]. The expression of ADRB2 in myocardial cells can strengthen muscular contractility, inhibit apoptosis in heart failure, and protect cardiac muscles form damages [25]. Therefore, damaged ADRB2 in COPD may reduce systematic ADRB2 expression, affect its myocardial protection in myocardial cells and thus lead to heart failure. The signaling cascade of ADRB2 cAMP/pka can have direct effects on the protein at neuromuscular joints and respond to adrenergic signals, thus maintaining the stability of the nervous system [26]; hence, the abnormal expression of ADRB2 would result in systemic myasthenia. In addition, ADRB2 can dilate the coronary artery and skeletal muscle [27] and assist the pancreas in secreting insulin [28].

LCK has been found to be mainly related to metabolic and cardiovascular diseases [29,30]. It is an essential constituent of the T-cell receptor (TCR) [31]. TCR identifies the antigen of pathogens [32] to generate an immune response, while LCK controls the initiation and proliferation of TCR signals [33]. Increasing evidence suggests that adaptive immunity dominated by T lymphocytes is involved in COPD pathogenesis, with an obvious increase in the number of CD8 T cells of COPD patients [34–36]. CD8 T cells can drive autoimmunity through cytotoxic activities and result in tissue injury [37–40]. In addition, the LCK of COPD patients seems to decline, which would lead to the dysfunction of T cells and inadequate infection response [33], thus resulting in recurrent airway infection of COPD patients and COPD aggravation [41]. As for the LCK regulation on the cardiovascular disease mechanism, some research reports showed that the LCK activation could play a role in protecting the heart during ischemia [42]. It is worthy of attention that T lymphocyte plays a key role in acute myocardial infarction and myocardial injury and may influence the clinical results of patients with coronary artery diseases [43]. The reason is that the immune response of patients with acute myocardial infarction is out of control and their regulatory T cells become less active, which results in more effector T cells, less anti-inflammatory cells, more severe inflammatory reactions and damaged organs. The findings of this study showed that obesity, lung cancer, diabetes and pulmonary hypertension were the four complications with the strongest correlation in the genetic and biological process with COPD aggravation. Both obesity and diabetes are metabolic complications, and moderate and severe COPD would increase the risk of diabetes [44]. The airway inflammation of COPD patients is the pathological and physiological foundation of diabetes. Airway obstruction is related to concentric obesity. Besides, patients with diabetes face a relatively higher risk (HR = 1.22) of suffering COPD than those without [45]. Diabetes also influences COPD prognosis. Compared with COPD patients free from diabetes, those with diabetes have a fatality HR value of 1.27 [46]. Another study showed that the mortality risk of COPD patients with diabetes was 15% higher than those without [47]. The close pathological and physiological tie between COPD and metabolic diseases is also revealed in the fact that such systemic inflammation markers as CRP, TNF-a and IL-6 would increase in diabetes and play an important role in the development of diseases. Smoking is one of the causes of inflammation, which explains the reason why smokers face a higher risk of diabetes than nonsmokers. Meanwhile, smoking and obesity bear a complex relationship with COPD aggravation: the adipose tissue is a critical place where cytokines (TNF-a and IL-6) form; adiponectin declines as obesity aggravates, which would enhance the resistance of insulin, circulate radicals and oxidative stress, and aggravate pulmonary inflammation [48–50]. Tissue hypoxia, smoking and bronchial obstruction accelerate the formation of the adipose tissue [51]. Inflammation markers (IL-6, TNF-a and CRP) correlate positively with weight, and the metabolic syndrome is related to pro-inflammation and coagulation [50]. The possible reasons why COPD complications aggravate COPD are as follows: obesity reduces pulmonary elasticity and weakens respiratory muscle functions through the non-enzymatic glycosylation of tissue protein; and as the strength of the inspiratory muscle disappears, pathological changes of the diabetic nerves damage the diaphragm [8].

The findings of this study showed that obesity, lung cancer, diabetes and pulmonary hypertension were the four complications with the strongest correlation in gene and biological process with COPD aggravation. Both obesity and diabetes are metabolic complications, and moderate and severe COPD would increase the risk of diabetes [44]. The airway inflammation of COPD patients is the pathological and physiological foundation of diabetes. Airway obstruction is related to concentric obesity. Besides, diabetes patients face a relatively higher risk (HR = 1.22) of suffering COPD than those without diabetes [45]. Diabetes also influences COPD prognosis. Compared with COPD patients free from diabetes, those with diabetes have a fatality HR value of 1.27 [46]. Another study showed that the mortality risk of COPD patients with diabetes was 15% higher than those without the disease [47]. The close pathological and physiological tie between COPD and metabolic diseases is also revealed in the fact that such systemic inflammation markers as CRP, TNF-a and IL-6 would increase in diabetes and play an important role in the development of diseases. Smoking is one of the causes of inflammation, which explains the reason why smokers face a higher risk of diabetes than nonsmokers. Meanwhile, smoking and obesity bear a complex relationship with COPD aggravation: adipose tissue is a critical place where cytokines (TNF-a and IL-6) form; adiponectin declines with a higher degree of obesity, which would enhance the resistance of insulin, circulate radicals and oxidative stress, and aggravate pulmonary inflammation [48–50]. Tissue hypoxia, smoking and bronchial obstruction accelerate the formation of adipose tissue [51]. Inflammation markers (IL-6, TNF-a and CRP) correlate positively with weight, and metabolic syndrome is related to pro-inflammation and coagulation [50]. The possible reasons why COPD complications aggravate COPD are as follows: obesity reduces pulmonary elasticity and weakens respiratory muscle functions through the non-enzymatic glycosylation of tissue protein; inspiratory muscle strength disappears; pathological changes to diabetic nerves injure the diaphragm [8].

A large number of epidemiological studies have demonstrated that there is a close connection between COPD and lung cancer. The findings show that the morbidity of COPD among lung cancer patients ranges from 40% to 70% [52,53] and that the annual morbidity of lung cancer among COPD patients is at least four times higher than the ordinary people [54]. The mechanism for COPD patients’ vulnerability to lung cancer has not been fully understood, but there are currently two main assumptions: first, the two share susceptibility genes; second, chronic inflammation plays a role in it. Smoking is a genetic risk factor shared by both diseases. The genome analysis of COPD and lung cancer patients showed that the susceptibility locus shared by the two diseases was found in multiple chromosomes, including 15q25, 4q31 and 6p21 [55]. The result shared by the analysis and this study is that both diseases have many common susceptibility genes which play roles in detoxification, the reconstruction of extracellular matrix, DNA restoration, cell proliferation and tumor inhabitation. Conversely, the mutation of the two genes at Locus 4q31 can protect smokers from COPD and lung cancer [56]. Besides, epigenetic alterations play a role in the pathological change to both lung cancer and COPD. For instance, DNA methylation, histone deacetylation, and protein phosphorylation have been proved to be involved in the pathological mechanism of these two diseases [57]. The second assumption is that chronic inflammation is mainly about epithelial-mesenchymal transition (EMT) which largely functions in carcinogenesis. Meanwhile, bronchitis also promotes EMT. Many pro-inflammatory reaction mechanisms are related to these two diseases, such as the activation of the transforming growth factor-b and the receptor tyrosine kinase/RaS access. In addition, the transcription factor NF-kB is the key protein in the pathogenesis and development of COPD, promoting the release of inflammation media. The genes regulating NF-kB are also found in tumor development and metastasis [58].

The main trigger of the occurrence of COPD complication of pulmonary hypertension is the long-term exposure to harmful stimulants, including cigarette and biofuel. Existing evidence has shown that airway stimulants contribute to chronic airway inflammation as well as pulmonary vascular change. The hypoxemia caused by COPD can result in vascular reconstruction and affect vascular dynamic and thus lead to pulmonary hypertension [59]. COPD-related hypoxemia is also attributed to secondary polycythemia, which causes an alteration to pulmonary vascular tension and then triggers pulmonary hypertension [60]. For COPD patients, an increase in the number of their leukocytes dominated by CD8+ lymphocytes would change the adventitia, restrain vasodilatation, and thicken the artery blood vessel intima of the pulmonary muscle. This indicates a correlation between pulmonary hypertension and vascular inflammation [61]. The inflammatory cytokines rising in COPD, such as IL-6, C-reactive protein and TNF-α, would also lead to ischemia, which further implies that inflammation can trigger pulmonary hypertension by changing the blood vessels of the pulmonary cycle [62,63].

After selecting the major genes indicating a correlation between COPD aggravation and complications, this study analyzed the relevance of biological processes between the two through gene enrichment. By selecting the biological paths of COPD aggravation and complications, we found that there are some differences in the percentage of shared genes and shared biological processes between metabolic syndrome and COPD. This difference also exists in cachexia, which may be caused by the following reasons: 1. The randomness of selecting COPD and COPD aggravation sample genes. 2. The number of shared genes and the number of shared biological processes are not necessarily linear. Due to the small number of metabolic syndrome data sets, the number of biological processes obtained by enrichment analysis is also small. 3. In shared biological processes, many processes involve less than 3 genes. In Figure 2, we deleted these biological processes in advance. At the same time,this study found that in the biological processes shared by COPD aggravation and complication, 471 biological paths were common between obesity and diabetes, accounting for 39.3% of the total number of biological paths of the two. Interestingly, other correlations found in this analysis included the ones between depression and diabetes and between sleep apnea and heart failure. Finally, the biological paths of some complications were all but irrelevant to that of other diseases, such as myasthenia and sleep apnea. In summary, understanding the relationship between COPD complications and aggravation in biological processes is important to comprehend the process of COPD aggravation.

## Conclusion

By analyzing the genes concerning COPD aggravation and complications, this study found that there was a genetic relevance between the two. As was shown in the study, the driver genes of COPD aggravation, including HP, ADRB2, LCK and CA1, could extensively regulate COPD complications, and that obesity, lung cancer, diabetes and pulmonary hypertension were the four complications that bore the closest relationship to COPD aggravation at the molecular level. The findings of this study were expected to provide a theoretical basis for prospective multi-target and multi-channel intervention in the occurrence and development of diseases and the future treatment of COPD and its complications.

## Data Availability

The data used to support the findings of this study are available from the corresponding author upon request.

## Research highlights

- Identified the molecular mechanism of COPD aggravation by an open database.
- Explored the relationship between COPD aggravation and its complications.
- Provided a direction for development of cures for COPD and its complications.
